# Texture Analysis of Chinese Dried Noodles during Drying Based on Acoustic–Mechanical Detection Methods

**DOI:** 10.3390/foods13020268

**Published:** 2024-01-15

**Authors:** Zhendong Cai, Zhenhua Wang, Min Zhang, Aojie Zhang, Guodong Ye, Shan Liang, Xin Ren

**Affiliations:** 1 Beijing Engineering and Technology Research Center of Food Additives, Beijing Technology and Business University, No.11 Fucheng Road, Haidian District, Beijing 100048, China; caizhendong58@163.com (Z.C.); zhwang@btbu.edu.cn (Z.W.); 15698993712@163.com (A.Z.); ygd1996btbu@163.com (G.Y.); liangshan@btbu.edu.cn (S.L.); renxin@btbu.edu.cn (X.R.); 2 Beijing Advanced Innovation Center for Food Nutrition and Human Health, Beijing Technology and Business University, No.11 Fucheng Road, Haidian District, Beijing 100048, China; 3 School of Food and Health, Beijing Technology and Business University, No.11 Fucheng Road, Haidian District, Beijing 100048, China

**Keywords:** Chinese dried noodles, texture characteristic, acoustic emission, mechanical testing

## Abstract

To better understand the textural transformation of Chinese dried noodles during the drying process, a convenient acoustic–force detection method was established. By comparing the breaking point, it was possible to determine the time-scale correlation between the force–displacement curves and acoustic spectrograms. The acoustic eigenvalues showed a consistent upward trend with the mechanical parameters during the drying process. With a wave crest reaching 152.8 dB and a signal maximum reaching 0.072, the structural stability of the dried noodles hardly induces a higher acoustic response. This suggests that the mechanical strength and rigidity of the dried noodles undergo minimal changes during this period. In comparison to the mechanical parameters, the acoustic eigenvalues accurately describe the changes in texture of dried noodles under various drying conditions, moreover, the sound threshold also provides a more effective response to the dried noodles’ structural strength threshold. Therefore, the acoustic detection method can be applied to assist the conventional mechanical measurement in the field of the texture evaluation of dried food.

## 1. Introduction

Noodles are a popular food worldwide and have had a profound impact on the food culture of countries such as China, Italy, and Japan. Chinese dried noodles are deeply embedded in China due to their simple storage conditions, ease of consumption, low price, and good taste [1,2]. Drying is a critical process in the production of Chinese dried noodles, and it is a complex process involving various phenomena such as protein denaturation, starch gelatinization, and glass transition, which leads to complex moisture transfer and structural changes occurring within the dried noodles during drying [3,4,5]. Therefore, the drying process induces a significant transformation in the texture of Chinese dried noodles. Controlling the texture transformation during drying is the key to ensuring the appearance quality, cooking quality, and eating quality of dried noodles.

The conventional approach to food texture research is predominantly focused on sensory and mechanical testing [6]. Sensory analysis plays a crucial role in quality management by enabling the visual assessment of a sample’s texture. However, the results are highly subjective to individuals [7]. The mechanical tests can provide quantifiable results, but they are often confined to specific mechanical features, which may not provide broader textural information on the product. When a force is applied to a brittle material for deformation, the external force causes the fracture of the brittle solid matrix, and the physical characteristics of the resulting acoustic signal depend on its textural and structural strength [8]. Consequently, sound analysis can serve as a reflection of the textural characteristics of the food fracture process [9,10,11]. In simultaneous testing, transducers are incorporated into the instrument, enabling the recording of the acoustic emissions generated during the crushing or shearing of the food sample. Noteworthy sound analysis applications include the evaluation of cracker crispness through acoustic envelope detectors and force and displacement measurements [9], investigation into the impact of water activity on the crispness of extruded rice [12], and the classification of puffed snacks’ freshness [13].

The methodology of acoustic–force coupling studies demands complex research conditions and instrumental support. Therefore, to effectively analyze and process the characteristics of the sound signal at the time of detection, this study designs a convenient acoustic–force combination method for assessing the texture of Chinese dried noodles. Compared to the existing combined acoustic–force detection methods, the use of Matlab 2019 software to analyze sound signals can expedite the research process. Additionally, attribute parameters can be modified to improve the precision and dependability of the study. By analyzing acoustic spectrograms and comparing force–displacement data with the associated physical properties, we aim to investigate the correlation between the texture of Chinese dried noodles and their acoustic properties and achieve an acoustic estimation of the physical properties of the texture characteristics of the Chinese dried noodles. Gaining insight into this relationship will facilitate a more rational analysis and evaluation of the textural transformation of the Chinese-dried-noodle drying process.

## 2. Materials and Methods

### 2.1. Materials

Wheat flour (moisture content 14.50% and protein content 12.87%) was purchased from Hebei Jinshahe Flour Manufacturing Co., Ltd. (Xingtai, China). Salt was purchased from China National Salt Industry Co., Ltd. (Beijing, China).

### 2.2. Methods

#### 2.2.1. Production of Chinese Dried Noodles

A total of 200 g of wheat flour was poured into a dough mixing machine (Model JHMZ 200, Dongfujiuheng, Beijing, China). Salt was added at a mass ratio of 1% (*w*/*w*) with the appropriate amount of water to achieve a final moisture content of 34% in the dough. Then, the dough was mixed for 4 min and sheeted on the noodle machine (Model JMTD 168/140, Dongfujiuheng, Beijing, China) at a roll gap of 1.5 mm. After resting at a temperature of 30 °C for 30 min in a self-sealing bag, the dough was sheeted at roll gaps of 1.2, 0.9, 0.7, and 0.5 mm, respectively. Finally, the dough sheet was cut into noodle streams 2 mm in width and 1 mm in thickness and dried in a constant temperature and humidity drying oven (Model BLC-250-III, Luxi, Beijing, China). For the drying test, a two-factor, three-level, full-permutation experimental design was adopted. Temperatures were set at 40, 60, and 80 °C, and relative humidity at 65%, 75%, and 85%. The drying time was 300 min, with a sampling interval of 50 min.

#### 2.2.2. Water Activity Measurement

Water activity (a_w_) was determined using a water activity meter (Model 4TE, AquaLab, Pullman, WA, USA). Five grams of Chinese dried noodles were cut and put into the sample cup, and the measuring temperature was set at 25 °C. Tendencies are fitted using Equation (1).

(1) $$Y=\left(Y0-C\right)*\exp\left(-K*X\right)+C$$

**where** Y0 is the Y value when X (time) is zero. C is the Plateau value at infinite times, expressed in the same units as Y. K is the rate constant, expressed in reciprocal of the X axis’ time units.

#### 2.2.3. Coupled Acoustic–Mechanical Detection

**Figure 1:**
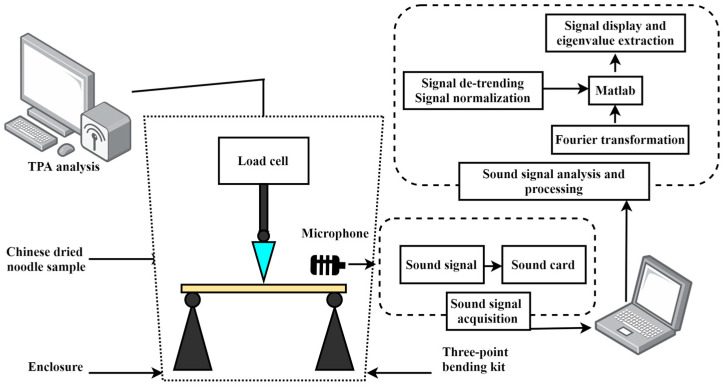
Schematic diagram of the coupled acoustic–mechanical test for Chinese dried noodles.

The primary component of the detection system comprises the three-point bending device of a textural analyzer (Model TMS-Pilot, FTC, Vienna, VA, USA), with sound acquisition facilitated by a sound card, which is illustrated in Figure 1. During the test, a probe is steadily pressed against the noodle until the sample is broken, and the acoustic signal is captured simultaneously from the sample rupture. The initial force applied is set at 0.05 N, and the testing speed is 48 mm/min. From the force–displacement data, several key parameters are derived, including the maximum shear force, the work done by the shear force, and the displacement. Combined with the data on the length, width, and thickness of the dried noodles, Young’s modulus is calculated using Equation (2).

(2) $$E=\frac{\sigma }{\varepsilon }=\frac{3FL}{2bh^{2}}*\frac{L^{2}}{6hD}=\frac{FL^{3}}{2bh^{3}D}$$

where σ is the stress (N/mm^2^); ε is the strain (mm/mm); E is the Young’s modulus (N/mm^2^); F is the rupture force (N); L is the length (mm); b is the width of the sample (mm); h is the thickness of the sample (mm); and D is the deformation in the middle of the sample (mm).

The sound signals are captured and imported into Matlab 2019 software. The signal is graphically represented in the frequency domain (via fast Fourier transform) and time–frequency domain (via short-time Fourier transform), and the relevant eigenvalues are extracted, with signal normalization and signal de-trending to obtain signal maximum, and the wave crest dynamic range is calculated using Equation (3).

(3) $$DR=20*{log}_{10}(\max\left(abs\left(x\right)\right)/min\left(abs\left(x\right)\right))$$

where DR is the dynamic range (dB) of the signal wave crest.

#### 2.2.4. Statistical Analysis

All data represent mean values derived from a minimum of three independent measurements. Microsoft Excel 2019 was used for data processing and SPSS 22.0 for statistical analysis. Duncan’s test was used to analyze the significant difference of the data at *p* < 0.05.

## 3. Results and Discussions

### 3.1. Water Activity

During the drying process, heat and mass transfer greatly impact the texture of Chinese dried noodles, with a strong correlation to both the moisture content and water activity [14]. However, it is difficult to describe the intricate dynamics of the water migration in noodles, and more attention is paid to the water activity in practical testing scenarios, which serves as a better predictor of texture change than moisture content [15]. Generally, higher water activity corresponds to a softer and tougher texture in noodles. This phenomenon can be attributed to the role of water as a plasticizer, and that the glass transition temperature of noodles increases with the decreasing water content, causing the dough to shift from a rubbery state to a glassy state, consequently enhancing its mechanical strength [16].

**Figure 2:**
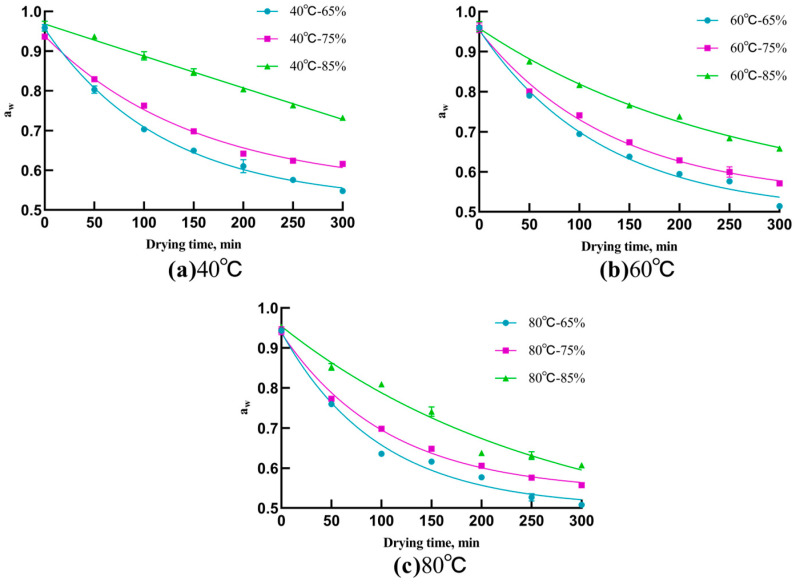
Fit curves of water activity of Chinese dried noodles under different drying conditions.

As shown in Figure 2, the water activity remained relatively high when the surface moisture of the Chinese dried noodles evaporated rapidly in the initial drying stage. As drying proceeded, the moisture inside the noodles began to migrate outward toward the surface, promoting a rearrangement and recombination of the internal structure of the noodles, which ultimately resulted in the development of a tough and elastic structure [17]. In the drying process, both temperature and humidity emerge as critical factors for ensuring the quality of dried noodles [18]. As shown in Figure 2c, elevated temperatures expedite the outward diffusion of moisture within the noodles, accelerating the decrease in water activity. This may lead to the formation of a hardened surface layer, resulting in an excessively hardened surface that can lead to an undesirable hard texture and a diminished taste [19]. The relative humidity plays a pivotal role in determining the rate of water evaporation from the noodle surface [17]. As shown in Figure 2, when the temperature remains constant, variations in the relative humidity directly impact the rate at which the internal moisture diffuses outward from the noodles, subsequently creating a decrease in water activity.

In summary, an increase in temperature accelerates the reduction of water activity, and an increase in relative humidity hinders the reduction of water activity. By manipulating the temperature and humidity conditions, diverse moisture states of dried noodles can be achieved, resulting in varied textures during the drying process. Such variations will be analyzed further through the application of acoustic–mechanical detection techniques.

### 3.2. Force–Displacement Curve and Time Domain Spectrogram

**Figure 3:**
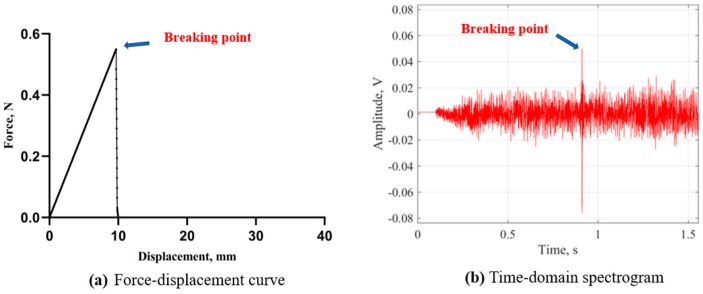
The mechanical and acoustic changes of Chinese dried noodles during the three-point bending process.

The three-point bending test is usually employed to simulate the chewing motion of the human mouth using a textural analyzer probe. The probe is linked to a computer and generates force–displacement curves, which can subsequently be analyzed through various mechanical parameters [20]. As shown in Figure 3a, when subjected to stress, the strain of Chinese dried noodles increased to a certain threshold, leading to the initiation of cracks in the noodles. Subsequently, the fracture area gradually decreased, nonlinear strain emerged, and stress concentration formed around the cracks, which resulted in a rapid increase in the stress to its peak value and finally led to the fracture of the dried noodle. Since cracks tend to expand in brittle materials, the noodles exhibited quick fracture behavior, and the maximum shear force of the transient fracture process was recorded by a computer. From Figure 3a, the crushing time of the Chinese dried noodles was short, leading to a steep slope in the force–displacement curve, which indicated that Chinese dried noodles have a high toughness due to their rapid alteration in strain strength when subjected to deformation.

Typically, investigations concerning food texture analysis depend on the force–displacement curve. However, each fracture occasion is concomitant with an up-and-down force change process as well as an associated acoustic event. The acoustic signals emitted during material fracture can also reflect its textural attributes [21]. As shown in Figure 3a, the time from the bending of the dried noodles to their fracture is exceedingly brief, making it a significant challenge to capture acoustic signals during this fleeting moment. Figure 3b shows the time-domain acoustic signal recorded during the three-point bending process of the dried noodles. This time-domain spectrogram reflects the variation in signal intensity over time [22]. As shown in Figure 3b, the sound card recorded the acoustic signal when the probe was in motion, and a conspicuous acoustic signal amplitude was observed at 0.91 s. Through a comparative analysis with the force–displacement curves in Figure 3a, this indicated that the dried noodles reached complete fracture upon reaching the maximum shear force value while simultaneously emitting a distinct acoustic emission signal. A temporal correlation was observed between the signal value at the moment of breaking and the maximum shear force value. At the breaking point, the physical characteristics of the acoustics and mechanics are both reflections of the dried noodles’ structure.

### 3.3. Acoustic Characteristic Spectrograms

Sound involves many parameters, including frequency, amplitude, magnitude, and time; thus, acoustic signal analysis is complex. Acoustic characteristic spectrograms can rapidly determine the information embedded within a particular segment or the entirety of a sound wave signal. This approach greatly streamlines the extraction of valuable and insightful eigenvalues from acoustic data. This is helpful in understanding the signal characteristics and provides a clear method to understanding the presence of the acoustic wave crest [22].

**Figure 4:**
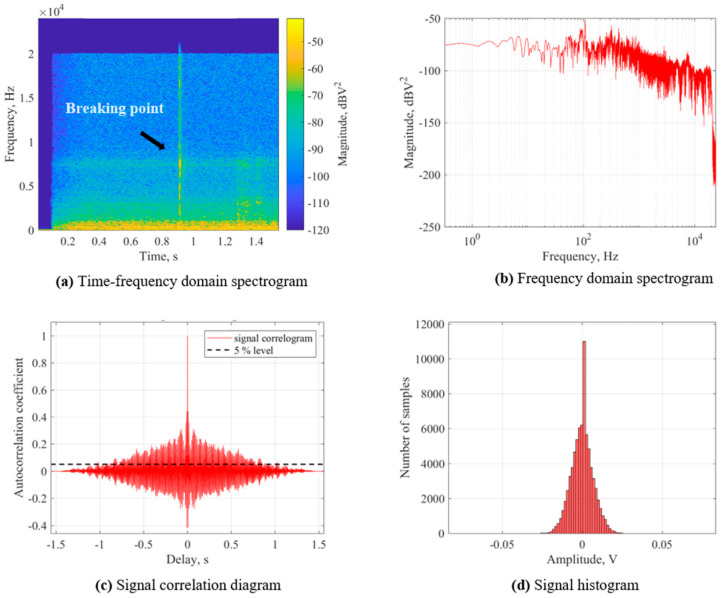
Acoustic characteristics of Chinese dried noodles during the three-point bending process.

Figure 4a is a time–frequency domain spectrogram, which shows the variation of the frequency and magnitude of the acoustic signal components during the bending process of the dried noodles. In Figure 4a, the breaking point of the dried noodles is clearly defined, and a maximum magnitude over −50 dBV^2^ is detected at this moment in time. Figure 4b is the frequency-domain spectrogram, which shows the magnitude distribution of the acoustic signal at different frequencies during the bending process of the dried noodles. The frequency of the bending process is concentrated between 10^2^ and 10^4^; a significant magnitude is observed in the frequency domain over 10^4^; the frequency intensity at this time represents the fleeting fracture process of the dried noodles. Thus, Figure 4a,b show that the acoustic signal at the breaking point had a greater frequency and magnitude, which was related to the loudness and clarity of the sound feature, indicating that the acoustic signal at the breaking point had a higher magnitude of volume and impact in its detection. Additionally, the higher the frequency and magnitude of the acoustic signal at the breaking point, the higher the loudness and clarity of the sound feature.

Figure 4c,d show the autocorrelation coefficients at various time delays and the proportion or probability density of signal samples with different amplitudes, respectively. The acoustic signals at the instant of the dried noodles’ rupture were analyzed in terms of frequency and waveform based on Figure 4c, and the autocorrelation coefficients were all lower than 0.01 (referring to the Supplementary Material). Combined with the distribution of the sample sizes in Figure 4d, the number of samples closest to 0 amplitude is the highest, indicating that the influence of noise in the signals was small and the signal-to-noise ratio was high. The autocorrelation time was between 0.9 s and 1.1 s (referring to the Supplementary Material), indicating that the waveform structure of each signal was consistent in the time series. It was shown that the acoustic signals captured during each drying process were consistent with the sample that we analyzed. The acoustic signals’ reliability was confirmed, enabling the assessment of the textural transformation throughout the drying procedure of Chinese dried noodles.

### 3.4. Mechanical and Acoustic Parameters

#### 3.4.1. Maximum Shear Force and Wave Crest

**Figure 5:**
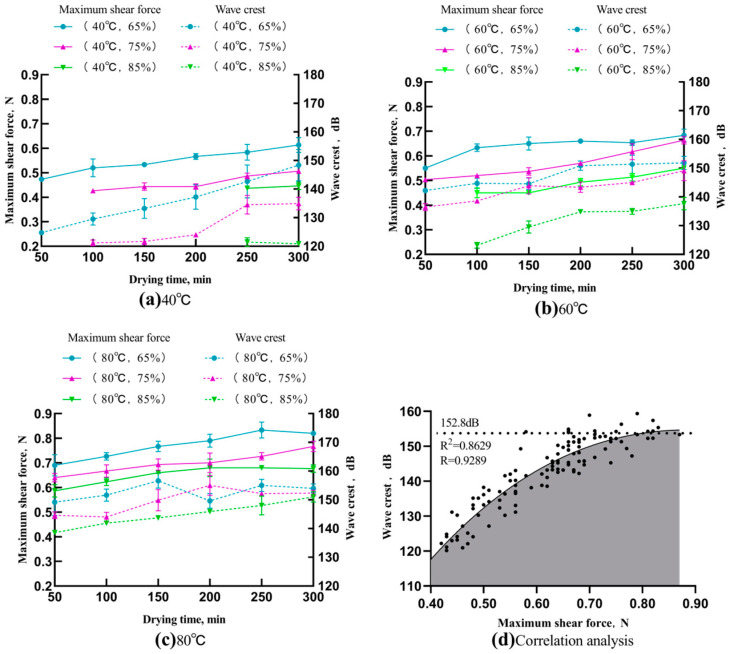
Variation curves of the maximum shear force and wave crest of Chinese dried noodles under different drying conditions.

The force–displacement curves and acoustic spectrograms were used to describe the mechanical and acoustic changes observed in the dried noodles during testing. To accurately characterize the texture of the dried noodles, a comparative analysis of their mechanical and acoustic strengths at the breaking point was conducted. The maximum shearing force refers to the highest point of shear stress that a material can withstand after reaching the yield point during the plastic deformation period. It is a critical reference to evaluate a material’s strength and toughness when subjected to shearing forces. In the food industry, it is frequently used to assess changes in the mechanical strength of tested samples. The term wave crest refers to the maximum value within an acoustic signal. The breaking point’s acoustic signal frequency and magnitude component intensity were directly related to the wave crest [23]. Figure 5a–c show an increasing trend in the maximum shear force of the dried noodles while they undergo the drying process, suggesting an improvement in their hardness and mechanical strength. Additionally, the acoustic signals captured at the breaking point of dried noodles displayed a gradual rise in their wave crest, indicating an increase in the frequency and magnitude components’ intensity. Therefore, the acoustic intensity and pitch of the dried noodles at the breaking point will increase and sharpen as the drying proceeds, indicating that the acoustic response will become clearer as the drying proceeds.

Figure 5a–c show how alterations in temperature and humidity affect both the shear force and wave crest trends. The shear force and wave crest increased with the temperature during the drying process, whereas they decreased with a relative humidity increase at a consistent temperature. This aligns with the findings on spaghetti that underwent varying temperature and humidity conditions. Spaghetti produced at high temperatures or low humidity displayed a greater rupture force [24]. This can potentially be attributed to substantial variances in the material volume shrinkage and water loss under different drying conditions, which result in the different structural strengths of the dried noodles [25]. As shown in Figure 5a–c, at a relative humidity of 65%, the shear force of dried noodles varied between 0.47 N and 0.61 N, 0.55 N and 0.68 N, and 0.69 N and 0.82 N at 40 °C, 60 °C, and 80 °C, respectively. The wave crest during the drying process at 40 °C showed a significantly greater increase of 23.59 dB, compared to only 9.08 dB at 60 °C and a mere 4.81 dB at 80 °C. As shown in the fitted curves presented in Figure 5d, the wave crest displays a pronounced upward trend with increasing shear force; however, the rising rate slows down. Nearly all the research on the relationship of sound to food texture concluded that the higher the sound wave and vibration’s signals, the bigger the structural strength or crispness’ increase in a given sample [26].

As shown in Figure 5a, when the drying temperature is lower, it can be seen that the maximum shear force of the dried noodles in the early drying period is lower, and the noodles exhibit weak mechanical strength. It may cause a visible change in texture as the shear force increases, resulting in a positive response of the acoustic signal at the breaking point. As the shear force is further increased, the mechanical strength of the noodles reaches a relatively higher degree, and the effect of the increased shear force on the texture is reduced. The increase in acoustic response is also reduced, and the acoustic signal’s wave crest reaches the threshold at an intensity of approximately 152.8 dB. Thus, the higher the wave crest, the higher the strength of the dried noodle structure, and as it approaches the threshold, the strength of the dried noodle product’s structure remains stable. It follows that the wave crest can be used as an acoustic indicator of the precise level of textural variation within dried noodles.

#### 3.4.2. Displacements and Shear Work

**Figure 6:**
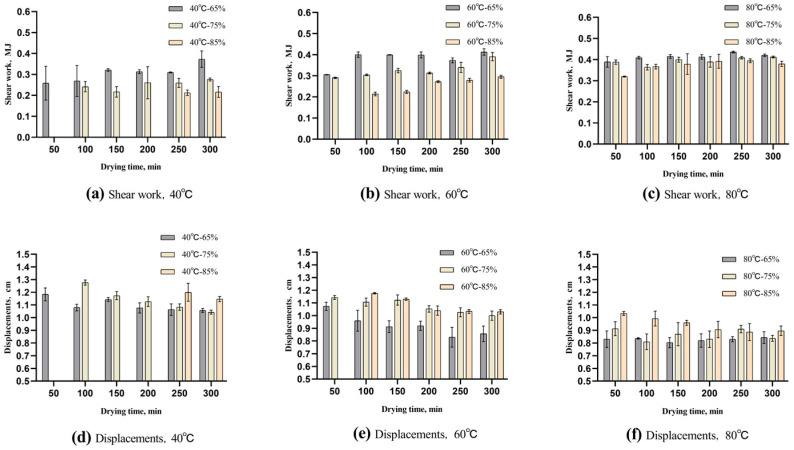
Variations of the shear work and displacements of Chinese dried noodles under different drying conditions.

Shear work serves as a measure of the energy that an object absorbs or releases during the deformation caused by a shear force. Typically, this energy comes from the thermal energy or elastic potential energy stored inside the material. Thus, when the dried noodles are bent and broken, the energy is released as sound waves, and the intensity of the wave crest can indicate its magnitude [26]. Figure 6a–c show an increase in shear work that occurs as the drying process progresses. Conversely, Figure 6d–f show a decrease in the displacement distance during drying. This phenomenon is attributed to surface hardening [27]. When dried noodles are bent, the core of the noodle experiences most of the deformation, while the outer shell remains intact for a certain period of time. The surface hardening during drying requires more shear force to break it. This increases the shear work and decreases the deflection of the dried noodles. Thus, the higher the strength of the dried noodle structure, the higher the shear work and the lower the displacements.

As shown in Figure 6a,d, at a relative humidity of 65%, the shear work of dried noodles varied from 0.258 MJ to 0.373 MJ and 0.390 MJ to 0.421 MJ at 40 °C and 80 °C, respectively. The displacements of the dried noodles varied from 1.14 cm to 1.06 cm and 0.83 cm to 0.81 cm at 40 °C and 80 °C, respectively. The conclusion of the drying process led to a rise of 0.115 MJ in the shear work at 40 °C, when exposed to 65% relative humidity. Meanwhile, there was also a decrease of 1.26 mm in the displacement distance. In contrast, the shear work only increased by 0.031 MJ at 80 °C, accompanied by a reduction of 0.2 mm in the displacement distance. This suggests that under high-temperature conditions, the dried noodles undergo a significant mechanical strength transformation early in the drying process. Therefore, it can be inferred that significant changes in the bending performance are present at 40 °C, while the changes at 80 °C are negligible.

Referring to the results analysis in Figure 5, the higher the wave crest, the higher the strength of the dried noodle structure. As shown in Figure 6a–c, the variation trend of the shear work was consistent with the maximum shear force results, and the wave crest also displays a consistent trend with the shear work. Thus, by comparing the results of the shear work and displacements, an apparent correlation between the wave crest and the bending performance of the dried noodles can be observed. Figure 6d shows the high bending performance at the low wave crest; in contrast, Figure 6f shows the weak bending performance at the high wave crest. Specifically, higher wave crests correspond to a weaker bending performance of the dried noodles; the weakest bending performance is observed at the threshold value.

#### 3.4.3. Young’s Modulus and the Signal Maximum

To further measure the texture transformation of the Chinese dried noodles during drying, the Young’s modulus and the acoustic maximum changes are analyzed based on the force–displacement curves and time-domain spectrograms. Young’s modulus serves as a measure of a material’s rigidity and its ability to undergo elastic deformation. It provides insight into a material’s resistance to deformation when subjected to tensile or compressive forces [28]. Converting time-domain signals to digital signals in the field of structural acoustics offers valuable insights into assessing the acoustic response of the structure, and can provide greater accuracy [29].

**Figure 7:**
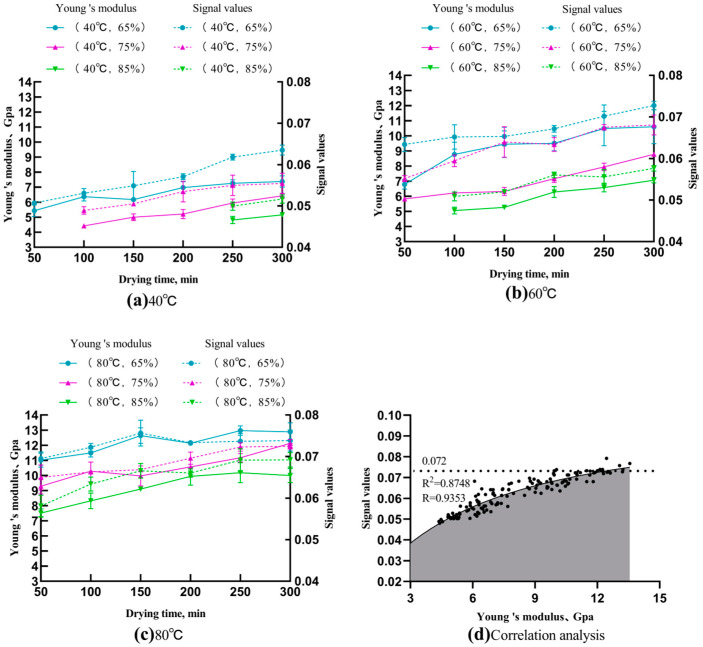
Variations of the Young’s modulus and acoustic signal maximum of Chinese dried noodles under different drying conditions.

Figure 7a–c show an increase in the Young’s modulus and signal maximum of dried noodles during the drying process. There is a clear and consistent increase in both Young’s modulus and the signal maximum under different drying conditions; an increased temperature increases the Young’s modulus and acoustic maximum during the drying process, whereas a higher relative humidity decreases the Young’s modulus and acoustic maximum at a constant temperature. This is consistent with the trends in the shear force and wave crest observed in Figure 5, which suggest an increase in the noodles’ rigidity as drying progresses. Furthermore, the acoustic response at the breaking point becomes more pronounced.

As shown in the fitted curves in Figure 7d, the signal maximum shows a pronounced upward trend with the increasing Young’s modulus; referring to Figure 5d, the rate of the signal maximum increase also slows down. Therefore, the higher signal maximum denotes the increased rigidity of the dry noodle. At low Young’s modulus levels, the noodles have weak rigidity, resulting in an apparent acoustic response when the Young’s modulus increases. When the Young’s modulus reaches a value of 10 GPa, the rigidity is already very high; if the Young’s modulus exceeds 10 GPa, the increase in the signal maximum becomes insignificant and the acoustic signal reaches a threshold near 0.072. This suggests that the rigidity of the dried noodles undergoes minimal changes during this period, thereby weaker differences are detected in the acoustic response.

## 4. Conclusions

Both the maximum shear force and Young’s modulus of dried noodles exhibited a consistent increase during the drying process, which led to an increase in the hardness and rigidity of the dried noodles. The wave crest and the acoustic signal maximum of the acoustic signals derived from the acoustic spectrograms also exhibited an upward trend. Through comparative analysis, the higher the crest, the higher the structure strength and the lower the bending performance of the dried noodles. A higher acoustic signal maximum corresponds to a higher rigidity of the dried noodles. When the wave crest is 152.8 dB and the signal maximum is 0.072, the sound signal reaches the threshold value, indicating that the structural strength of the dried noodles reaches its critical degree. Consequently, the eigenvalues derived from the acoustic signals can assist in mechanical detection methods, facilitating a comprehensive evaluation of the textural attributes of dried noodles. In the food industry, there has been limited research on the relationship between acoustic and textural characteristics, and, in the future, acoustic metrics should be further differentiated and defined, eventually leading to universal standards. The coupled acoustic–mechanical detection method employed in this study not only provided valuable insights but also presented a promising innovative approach for the study of food texture.

## Data Availability

Data is contained within the article or Supplementary Material.

## Supplementary Materials

The following supporting information can be downloaded at: https://www.mdpi.com/article/10.3390/foods13020268/s1, Table S1: Autocorrelation coefficient and autocorrelation time results based on acoustic characteristic spectrograms.
