# Supplementary material for: Texture Analysis of Chinese Dried Noodles during Drying Based on Acoustic–Mechanical Detection Methods
Source: Foods. 2024 Jan 15;13(2):268. doi: 10.3390/foods13020268 (PMC10815251; doi:10.3390/foods13020268)
Supplement: Supplementary file 1 [file foods-13-00268-s001.zip › foods-2733312-supplementary.pdf]

Table S1. Autocorrelation coefficient and autocorrelation time results based on acoustic characteristic spectrograms

drying temperature/℃	Drying time/min	%RH	autocorrelation coefficient	Autocorrelation time/s
40℃	50	65	0.006767	1.04±0.03[a]
		75	——	——
		85	——	——
	100	65	0.007124	1.01±0.06[a]
		75	0.007998	1.02±0.03[a]
		85	——	——
	150	65	0.007721	1.04±0.03[a]
		75	0.006983	1.07±0.03[a]
		85	——	——
	200	65	0.007533	0.97±0.06[a]
		75	——	1.09±0.03[a]
		85	——	——
	250	65	0.006401	0.91±0.01[a]
		75	0.007114	1.04±0.01[a]
		85	0.006762	1.08±0.02[a]
	300	65	0.007536	0.93±0.03[a]
		75	0.006734	1.04±0.01[a]
		85	0.006946	1.08±0.02[a]
60℃	50	65	0.007351	0.98±0.05[a]
		75	0.007166	1.02±0.05[a]
		85	——	——
	100	65	0.00723	0.94±0.01[a]
		75	0.00725	1.02±0.01[a]
		85	0.007219	1.04±0.03[a]
	150	65	0.007599	0.94±0.01[a]
		75	0.007381	1.03±0.05[a]
		85	0.007347	1.04±0.06[a]
	200	65	0.007497	0.95±0.03[a]
		75	0.007179	0.99±0.04[a]
		85	0.006119	1.04±0.03[a]
	250	65	0.007871	0.95±0.03[a]
		75	0.006724	0.95±0.02[a]
		85	0.007317	1.04±0.01[a]
	300	65	0.007143	0.94±0.03[a]
		75	0.006126	0.92±0.01[a]
		85	0.006734	1.01±0.07[a]
80℃	50	65	0.007465	0.96±0.03[a]
		75	0.007356	0.95±0.01[a]
		85	0.007363	0.97±0.02[a]
	100	65	0.007392	0.94±0.00[a]
		75	0.007451	0.95±0.01[a]

		85	0.007477	0.96±0.06[a]
		65	0.007655	0.95±0.03[a]
	150	75	0.007443	0.94±0.03[a]
		85	0.007385	0.94±0.01[a]
		65	0.007237	0.91±0.03[a]
	200	75	0.007228	0.95±0.03[a]
		85	0.007232	0.96±0.03[a]
		65	0.006394	0.95±0.01[a]
	250	75	0.007271	0.95±0.01[a]
		85	0.006784	0.95±0.03[a]
		65	0.007486	0.96±0.02[a]
	300	75	0.007245	0.94±0.03[a]
		85	0.007363	0.93±0.02[a]
